# Crosstalk between Myopia and Inflammation: A Mini Review

**DOI:** 10.7150/ijms.94826

**Published:** 2024-06-03

**Authors:** Jizhao Xin, Bo Bao, Jinpeng Liu, Zhongyu Ma, Miao Zhang, Hongsheng Bi, Dadong Guo

**Affiliations:** 1 Shandong University of Traditional Chinese Medicine, Jinan 250002, China.; 2 Affiliated Eye Hospital of Shandong University of Traditional Chinese Medicine, Jinan 250002, China.; 3 Shandong Provincial Key Laboratory of Integrated Traditional Chinese and Western Medicine for Prevention and Therapy of Ocular Diseases; Shandong Academy of Eye Disease Prevention and Therapy; Medical College of Optometry and Ophthalmology, Shandong University of Traditional Chinese Medicine, Jinan, 250002, China.

**Keywords:** myopia, inflammation, allergic reaction, inflammatory factors

## Abstract

Myopia represents a significant public health concern worldwide, particularly affecting the ocular health of children and adolescents. The escalating prevalence of myopia in recent years underscores its urgency as a health issue among this demographic. Research indicates a profound connection between the onset of myopia, inflammatory processes and fibrosis. Individuals with inflammatory conditions like allergic conjunctivitis, choroiditis, systemic lupus erythematosus, and diabetes exhibit a heightened susceptibility to myopia. Conversely, myopic patients are at an increased risk of developing ocular inflammatory disorders, notably idiopathic multifocal choroiditis. We postulate that the expression of inflammatory markers, including NF-κB, TGF-β, IL-1β, IL-6, IL-8, and TNF-α, may contribute to the chronic inflammatory state observed in myopia. This paper highlights a substantial correlation between myopia and inflammation, suggesting the potential efficacy of anti-inflammatory agents in managing inflammation and slowing myopia progression.

## Introduction

Myopia is an enlargement of the vitreous chamber of the eye, which results in an elongation of the eye axis, leading to the concentration of light rays from distant objects in front of the retina and resulting in a blurred image. In recent years, the prevalence of myopia has risen dramatically globally, and in particular, the "coronavirus disease 2019" (COVID-19) epidemic has adversely affected myopia progression^1^. It is estimated that more than half of the world's countries will have more than 50% of their populations suffering from myopia by 2050^2^,^3^, and the prevalence of high myopia is estimated to be around 20%. High myopia exacerbates the risk of ocular pathologic changes such as glaucoma, retinal detachment, and macular and choroidal neovascularization, with a projected seven-fold increase in visual impairment^4^. The impact of myopia is far-reaching and affects people's lives medically, socially, and economically, leading to a decline in their quality of life^5^.

The cause of myopia remains a mystery, but a great deal of evidence suggests that genetic factors and environmental exposures are critical for localized eye growth and refractive development^6^. Myopia is characterized by scleral thinning and localized dilatation of the posterior sclera^7^. Clinical and experimental studies have shown that eye elongation is associated with changes in the extracellular matrix (ECM) and remodeling of the scleral connective tissues^7^,^8^. The regulation of ECM composition and scleral remodeling is determined by genes, environmental conditions, and individual behavioral factors^9^. At present, the exact biological means by which environmental factors influence refraction in the human eye remains obscure; however, it is suggested that inflammation is a factor in susceptibility to myopia^10^. A cohort study conducted by Lin *et al.* demonstrated that individuals with autoimmune disorders such as type 1 diabetes mellitus (T1DM) and systemic lupus erythematosus (SLE) are more susceptible to myopia^11^. Lin *et al.*^11^ observed elevated inflammatory marker levels, including c-Fos, nuclear factor-kappa B (NF-κB), interleukin (IL)-6, and tumor necrosis factor (TNF)-α in myopic eyes. However, treatment with atropine and cyclosporine A (CSA) reduced these inflammatory factors and inhibited myopia progression, indicating that both atropine and CSA exert a similar controlling effect on myopia progression^11^. Based on clinical and experimental evidence, inflammation appears to play a significant role in myopia progression.

Currently, most research that promotes myopia progression focuses on environmental and genetic aspects. However, few studies are involved in inflammatory diseases. In this paper, we analyze the effects of multiple inflammatory-type diseases on the progression of myopia to address the underlying mechanisms, assisting in the treatment and prevention of myopia.

## 1. Inflammation induces myopia progression

Several ocular degenerative diseases which may affect choroidal and retinal neurons are associated with myopia. Epidemiologic data suggest that children with allergic conjunctivitis (AC) are at greater risk for myopia^12^. Wei *et al.*^12^ demonstrated that mast cell degranulation induces inflammation of the ocular surface, which alters the corneal tight junctions and contributes to corneal secretion of inflammatory cytokines, leading to retinal inflammation and myopia progression. Lin *et al.*^11^ found that patients with inflammatory diseases such as SLE (3.5%), uveitis (5.2%), and T1DM (7.9%) had a higher prevalence of myopia than patients without such diseases. These findings demonstrate experimentally and clinically that inflammation has the potential to contribute to the development of myopia.

### 1.1 Allergic conjunctivitis-induced myopia

Allergic conjunctivitis (AC) is an abnormal immune hypersensitivity reaction to environmental allergens and is a source of inflammation affecting the ocular surface^13^. The inflammatory response to AC is divided into two main phases. The early response is an Ig-E-mediated hypersensitivity reaction, in which the patient's exposure to allergens results in the binding of specific Ig-E to the sensitized mast cells, and activation of mast cells induces an increase in secretion of histamine, tryptase, prostaglandins, and leukotrienes in the tears, and a concurrent increase in secretion of histamine, tryptase, prostaglandins, and leukotrienes in the tear fluid. Mast cell degranulation also induces the activation of vascular endothelial cells, which then express chemokines and adhesion molecules such as ICAM and VCAM. The immune mechanism of allergic conjunctivitis is characterized by mast cell degranulation, immunoglobulin Ig-E, and immune responses by T lymphocytes. The late response is regulated by activated T cells expressing and secreting chemokines, monocyte chemotactic proteins, IL-8, eosinophil chemokines, and macrophage inflammatory proteins 4-6 hours after the reaction is initiated. These factors initiate the recruitment of inflammatory cytokines in the conjunctiva, leading to a delayed ocular response. Inflammatory cells such as neutrophils, eosinophils, lymphocytes, and macrophages are histological features of conjunctival infiltrative allergic conjunctivitis, and chronic allergic conjunctivitis may lead to remodeling of ocular surface tissues^13^. Meanwhile, both allergic diseases and myopia are chronic diseases with long-term progression. Epidemiological analysis by Wei *et al.* showed that the myopia rate in patients with allergic conjunctivitis was 2.35 times higher than that in patients with nonallergic conjunctivitis^12^.

**Figure 1:**
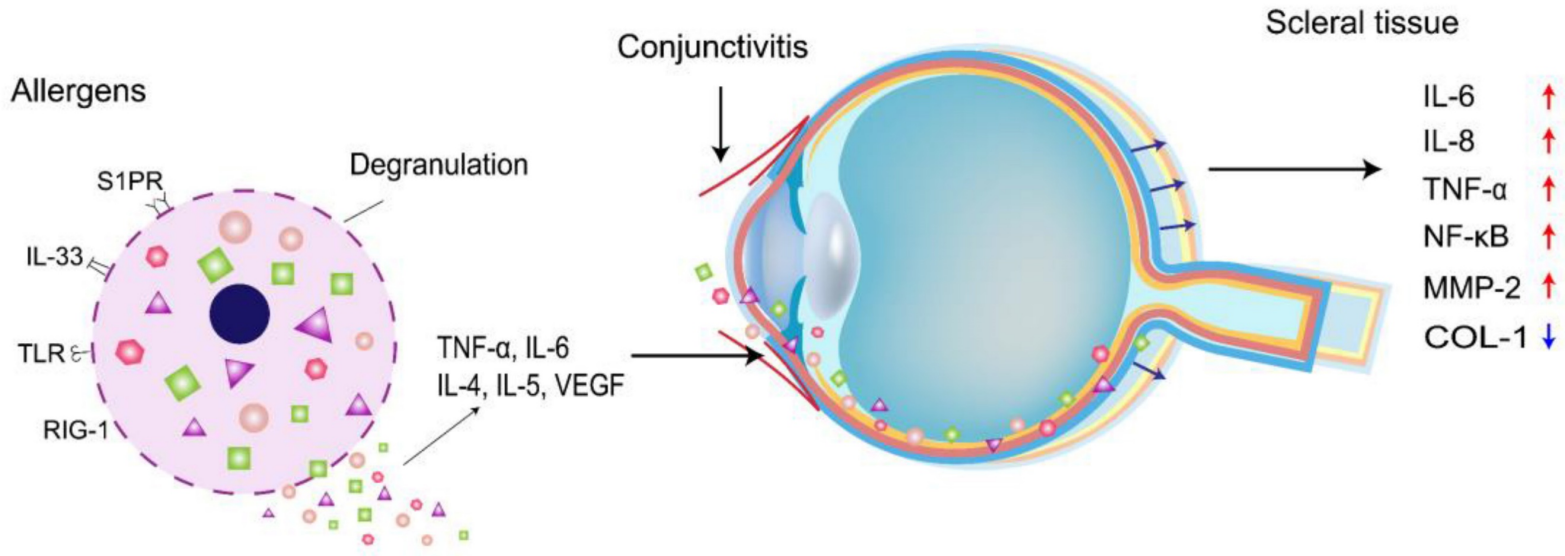
Schematic representation of allergic conjunctivitis modulating inflammatory cytokines to promote myopia progression.

The sclera is the structural framework that maintains the shape and integrity of the eye. In myopia, the depth of the vitreous cavity increases, and the length of the ocular axis elongates. To adapt to this inappropriate ocular axis elongation, the thickness of the sclera is reduced, thereby increasing its stretchability, and the thinning process is related to decelerated synthesis, accelerated degradation of the collagen skeleton, and altered composition of the ECM^14^. Collagen type 1 (COL-1) is the primary ECM, accounting for approximately 90% of the dry weight of the sclera. Different isoforms of proteases are known to mediate ECM degradation and scleral thinning, and MMP-2 is one of the enzymes upregulated in this process^15^. An increase in MMP-2 decreases the accumulation of COL-1, which weakens the scleral skeleton, increases the length of the ocular axis, and thins the sclera. In addition, MMP-2 knockdown significantly inhibits the reduction of type I collagen (COL1α1) scleral α1 chain accumulation during myopia^16^. Thus, scleral thinning in myopic eyes is due to reduced COL-1 and ECM degradation. Wei *et al.* showed that the permeability of the conjunctival blood-water barrier increased in patients with AC. Therefore, ocular surface inflammation can also increase intraocular inflammatory factors' expression levels^12^. Inflammatory cytokine levels of IL-6, IL-8, and TNF-α are elevated in AC patients^17^, where the expression levels of IL-6 and TNF-α are consistent with the levels of IL-6 and TNF-α in patients with myopia. IL-6 activates the NF-κB pathway, which stimulates the production of TNF-α in the cornea^18^. Continued inflammation of the cornea affects the expression of intraocular inflammatory cytokines, and intraocular IL-6, IL-8, and TNF-α in turn activate NF-κB and increase the expression of MMP-2, an important molecule in ocular tissue remodeling, which breaks down collagen, leading to scleral remodeling and contributing to myopia progression (Figure 1). It has been proposed that allergic conjunctivitis may be in a pro-inflammatory state, affecting myopia progression due to the surge of pro-inflammatory cytokines and the decrease of anti-inflammatory cytokines. Mimura *et al.*^20^ also found that AC patients with specific Ig-E to indoor antigens had higher myopia than those without allergies.

### 1.2 Ciliary inflammation-induced myopia

Uveitis often precedes various systemic autoimmune disorders affecting the uvea, specifically the iris, ciliary body, and choroid. A rare clinical manifestation is unilateral ciliary inflammation leading to myopia, distinct from the typical bilateral accommodative spasms in pseudomyopia. Umar Ijaz *et al.* described a case where a young man presented with acute anterior uveitis accompanied by unilateral myopia. Following uveitis treatment, the patient's visual acuity and refraction improved. This subtle cellular reactive myopia might be overlooked, as it is potentially linked to zonular fiber relaxation due to exudates on the ciliary body, thus altering lens convexity.

**Figure 2:**
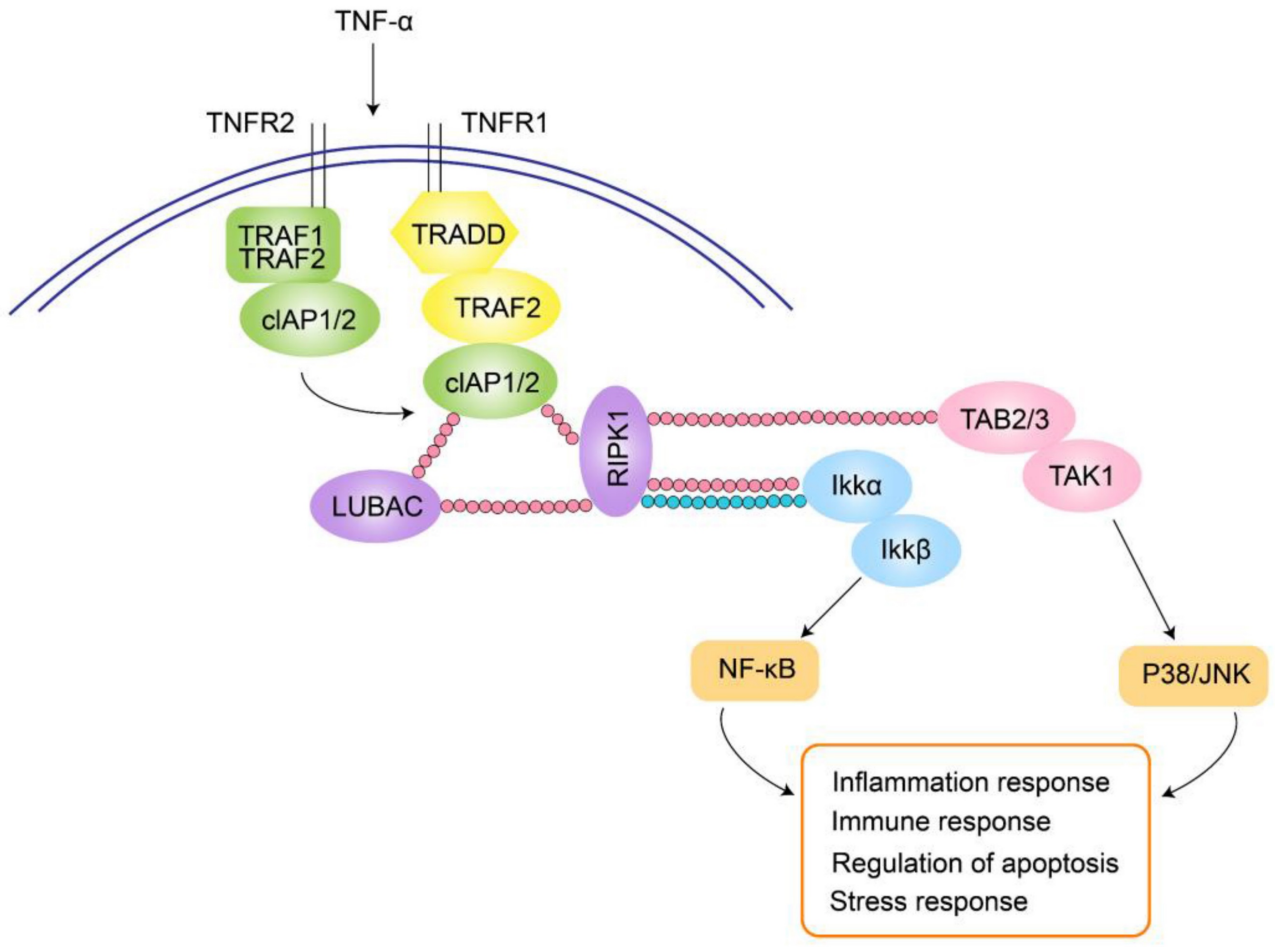
Schematic diagram of uveitis modulating inflammatory cytokines to promote myopia progression.

In patients with ciliary body inflammation, TNF-α expression increases in both serum and aqueous humor. Intraocular pigment epithelial cells produce TNF-α and matrix metalloproteinases, then TNF-α is converted from its transmembrane form to a soluble, circulating form within the eye. These cells also express TNF receptors 1 (TNFR1) and TNFR2, mediating TNF-α-induced intraocular inflammation. TNF-α binding to TNFR1 initiates a signaling cascade involving TRADD, RIPK1, LUBAC, TRAF2, and cIAP1/2. TNFR2 directly interacts with TRAF1/2, triggering cIAP1/2 recruitment and subsequent binding to the TNFR1 signaling pathway. cIAPs attach linear polyubiquitin chains to RIPK1 and recruit LUBAC, enabling the assembly of TAK1 and IKK complexes. These complexes mediate the p38/JNK and NF-κB pathways, regulating tissue regeneration, cell proliferation, and inflammation (Figure 2). In addition, experimental studies also indicate a close association between the NF-κB pathway and myopia progression.

### 1.3 Chronic uveitis associated with juvenile idiopathic arthritis induced myopia

Juvenile idiopathic arthritis (JIA) is the most common systemic disease associated with chronic anterior uveitis (CAU) in children, accounting for 47% of all types of uveitis in children^24^. JIA is a progressive rheumatic disease usually presenting in childhood, and its pathogenesis is characterized by chronic inflammation of the joints of unknown etiology. Based on the course of the disease, the International League of Associations for Rheumatology (ILAR) has identified seven subtypes^25^, of which the enthesitis-related arthritis (ERA) and psoriatic arthritis (PsA) subtypes are at high risk for acute and recurrent uveitis.

The relationship between refractive error and JIA has been evaluated in 65 adult patients at Hornbæk Physical Medicine Hospital. Patients with JIA may experience ocular involvement in both eyes during the early stages of the disease, although ocular inflammation is often asymptomatic at this point. It was observed that myopia typically develops after an average duration of 4.22 years. The refractive errors observed in these patients ranged from -8.12 D to +6.5 D, with a mean standard deviation of -0.64 D and a median of 0^26^. Notably, patients with JIA exhibited significantly higher myopic refractive errors in comparison to controls, indicating a potential link between JIA and myopia progression.

Complications arising from JIA-associated uveitis include macular cystoid edema, band keratopathy, amblyopia, ciliary body inflammation, and secondary glaucoma, all of which can contribute to vision loss^24^. Adolescents are particularly vulnerable as their eyes are in an active growth phase. The sclera, being more susceptible during this period, can be affected by the compressive forces of elevated intraocular pressure resulting from uveitis, leading to a weakening scleral connective tissue. Additionally, elevated inflammatory cytokine levels, such as TNF-α and IL-6 in the aqueous humor of uveitis patients, may contribute to myopia progression^23^. The signaling pathways involved in the action of these inflammatory factors are shown in Fig. 2.

### 1.4 Dental caries and periodontitis promote myopia

Dental caries and periodontal disease are diseases that affect many people worldwide, and risk factors, including physical inactivity and nutritional deficiencies, may be associated with an increased incidence of dental caries and myopia^27^,^28^. Systemic inflammation is associated with periodontitis^29^, and active dental caries^30^ may also be associated with various eye diseases.

**Figure 3:**
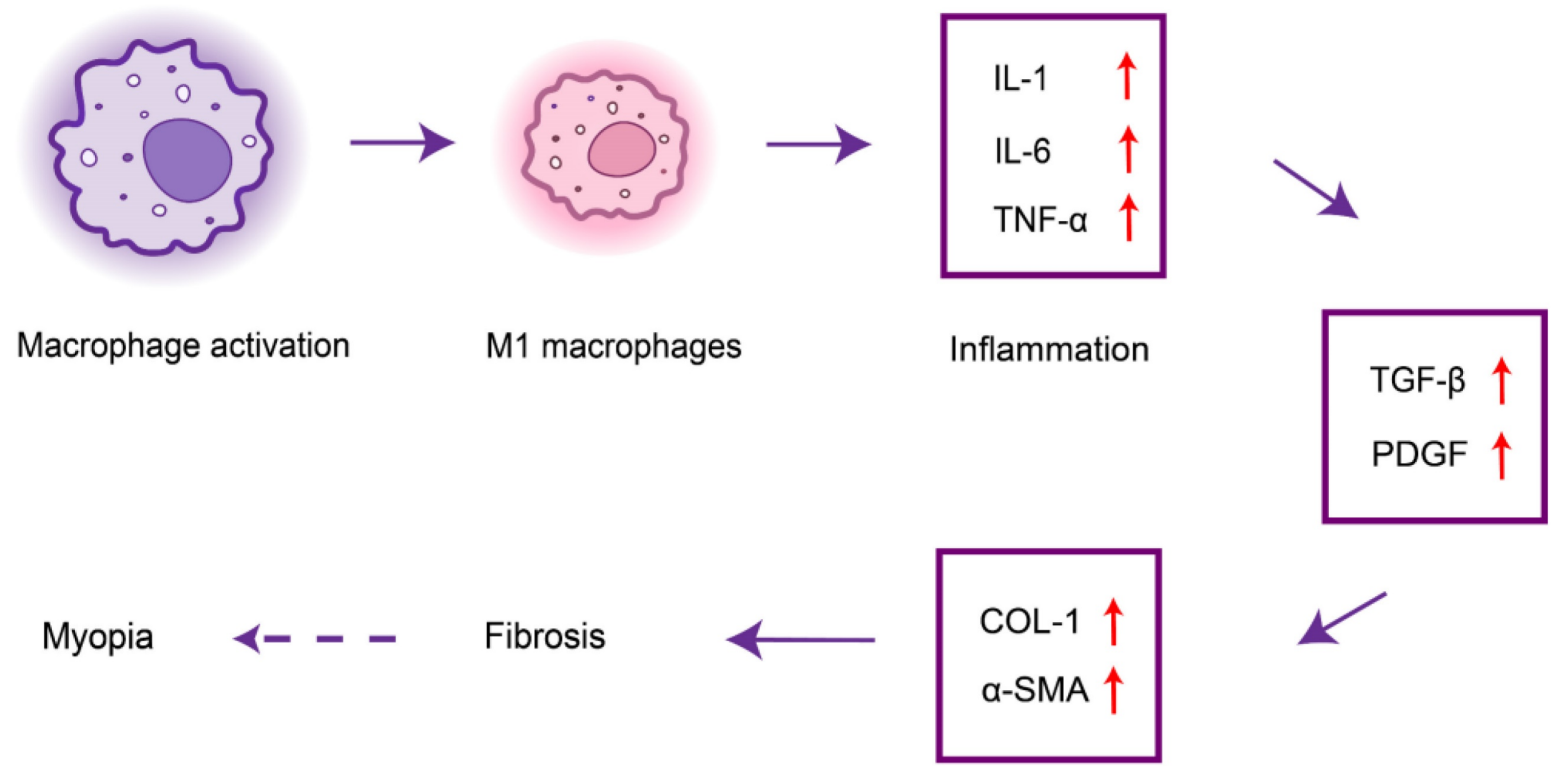
Schematic diagram of dental caries and periodontitis regulating inflammatory cytokines to promote myopia progression.

Since the 1970s, researchers have explored the correlation between myopia and dental caries. Tsai *et al.* found that the inflammatory state of the teeth in the near oral cavity may be associated with myopia^31^. Poor oral hygiene leads to bacterial growth in the teeth^32^, which can activate dental caries, leading to tooth destruction and systemic inflammation. Treating active dental caries can greatly reduce the effects of systemic inflammation on the fibrosis of ocular tissues, thereby reducing the risk of myopia^33^,^34^. Oral biofilms stimulate the secretion of pro-inflammatory cytokines (e.g., TGF-β, IL-1, IL-6, and TNF-α) from host immune cells, which can lead to the destruction of periodontal tissues^35^,^36^, and these cytokines play a crucial role in inflammation and fibrosis. Localized inflammation can adversely affect systemic health^37^. Macrophages are the main source of platelet-derived growth factor (PDGF) and can lead to collagen overproduction and fibrosis along with TGF-β. In addition, α-SMA and COL-1 are known markers of myofibroblastogenesis, and both may promote fibrosis of ocular tissues such as the ciliary muscle, affecting ocular accommodation and leading to myopia progression 38 (Figure 3).

### 1.5 Diabetes and myopia

Diabetes mellitus (DM) causes ocular problems that can lead to cataracts, glaucoma, retinopathy, optic neuropathy, uveitis, and even loss of vision, which adversely affects ocular health. Both the Barbados Eye Study^39^ and the Los Angeles Latino Eye Study^40^ have shown that diabetes is a risk factor for myopia and T2DM is an inflammatory disease accompanied by elevated levels of TGF-β, IL-1β, IL-6, TNF-α, and fibroblasts^41^.

The surface curvature of the lens is greater in DM patients than in non-DM patients^42^, and osmotic fluid shifts due to DM-induced hyperglycemia can cause refractive changes, leading to lens hydration and even myopia. The findings of Jacobsen N *et al.*^43^ support the association of myopia with impaired metabolic control, stating that the enhanced scleral growth may be due to elevated levels of insulin and insulin-like growth hormones. Giri *et al.* 44 showed that high glucose levels contribute to developing diabetes-related eye disease by stimulating the polyol pathway, promoting late glycosylation end products, activating protein kinase C, increasing oxidative stress, and increasing inflammatory pathways. There is growing evidence that both myopia and T2DM may be associated with pathophysiologic pathways mediated by insulin resistance, and the role of insulin and insulin-like growth factors on refractive changes cannot be ignored^45^.

**Figure 4:**
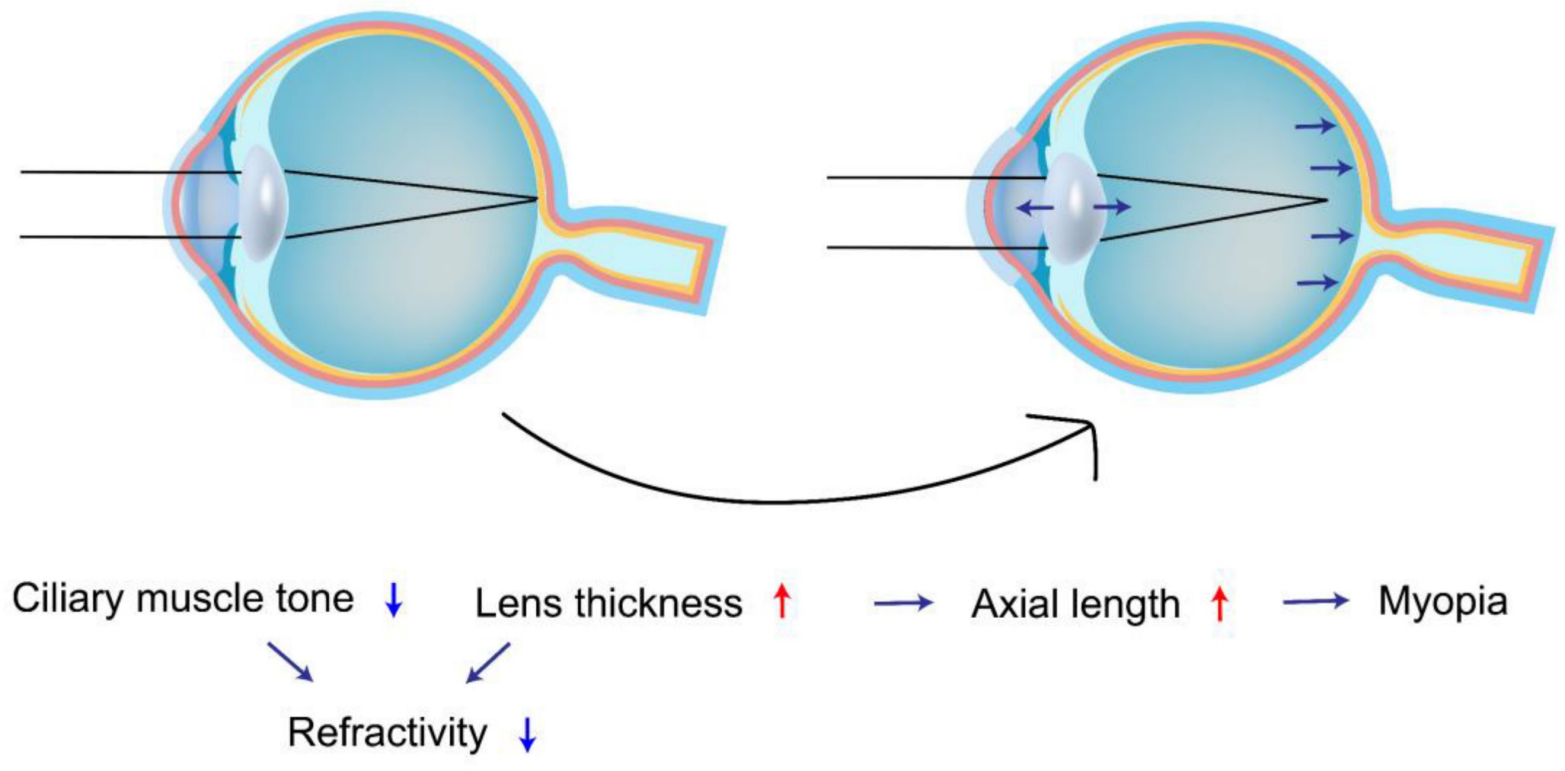
Schematic diagram of diabetes affecting refractive interstitial changes in the eye.

A study of the lens by Wiemer *et al.*^46^ found that the lens is composed of a nucleus and three cortical regions and that patients with T1DM had a significantly thicker lens and a more convex crystalline lens in all structures compared to healthy control subjects, which supports the idea that the thickening of the lens is the result of extracellular overhydration. Meanwhile, Wiemer *et al.*^46^ also found that patients with T1DM had a smaller magnitude of lens accommodation, less ciliary muscle contraction, and a significantly lower equivalent refractive index, which implies that the eyes of patients with T1DM are less tense and thus more susceptible to myopia (Figure 4).

Lin *et al.*^47^ conducted a study revealing that patients under 40 years old with Type 1 Diabetes Mellitus (T1DM) face a heightened risk of myopia and associated illnesses compared to those with Type 2 Diabetes Mellitus (T2DM). Notably, females in this age group are at an even greater risk than males. Furthermore, both T1DM and T2DM patients are more prone to myopia and related conditions than non-diabetics^48^. Specifically, T2DM patients aged 40-59 years exhibit a higher prevalence of myopia than non-diabetics or T1DM patients. It's worth noting that visual acuity in these patients fluctuates with blood glucose levels, and refractive errors in intraocular lenses (IOL) can cause blurred vision^49^. However, among patients aged 60 and above, no discernible differences in myopia or related disorders were observed between those with T1DM, T2DM, and non-diabetics. The decreased risk of myopia appears to stem from aging rather than diabetic modulation. The risk of myopia decreases with natural aging among patients with DM, and men have a significantly lower risk of myopia than women.

Adolescents find themselves in a pivotal growth and development phase where the functionality of the lens and ciliary muscles remains unstable. During this developmental stage, eye tissue is particularly fragile and exceptionally susceptible to external disruptions, potentially leading to the onset of myopia. Therefore, diabetic patients under the age of 18 years are particularly susceptible to myopia and astigmatism due to diabetes. Li S. M *et al.*^50^ showed that in Chinese children aged 7-14 years old, the lens thickness (LT) usually decreased by 0.19 mm and that the increase in myopia was closely related to an increase in the axial length (AL) and LT as well as a decrease in the radius of corneal curvature. In an ophthalmologic examination study of 54 patients with T1DM and 53 healthy subjects under the age of 18 years, Xiao *et al.*^51^ found that LT in patients with T1DM was significantly greater than that of the reduction in anterior chamber depth (ACD) compared to the control group. Nevertheless, there was no significant difference in AL, so the higher the value of LT in patients with T1DM, the higher the risk of myopia. Therefore, it is important for adolescent diabetics, especially those with poor glycemic control, to have regular checkups and prompt treatment of myopia.

### 1.6 Systemic lupus erythematosus and myopia

Chronic autoimmune inflammation and systemic lupus erythematosus (SLE) may affect multiple organs and systems. Under normal physiological conditions, apoptotic cells undergo phagocytosis. Failure to phagocytose these cells results in the release of self-antigens from their contents, potentially triggering autoimmunity^52^. SLE is characterized by a deficiency in classical complement proteins, which impairs the phagocytosis of immune complexes, apoptotic cells, pathogens, and foreign bodies. Lymphocyte apoptosis in SLE patients may play a crucial role in disease pathogenesis. Typically, apoptotic vesicles containing nucleosomes and organelles can be promptly cleared by phagocytes upon formation. However, an increase in apoptosis can lead to an excess release of nucleosomes from apoptotic cells. These nucleosomes are a source of nuclear antigens that stimulate the immune response, possibly inducing the production of anti-DNA and anti-histone antibodies. Subsequently, T cells and B cells target these substances, generating autoantibodies that initiate an autoimmune reaction and the formation of immune complexes. Consequently, SLE involves the deposition of these antigen-antibody complexes in various tissues, ultimately resulting in cell death and inflammation^52^.

Different mechanisms, such as immune complex deposition, vasculitis, thrombosis, and other antibody-related mechanisms, can lead to ocular manifestations caused by SLE^53^. The literature suggests that altered lens curvature or anterior displacement of the lens-iris septum leads to acute myopia^54^. Immunosuppressive agents can treat inflammatory types of SLE-associated visual impairment, such as retinopathy or ciliary body inflammation^55^. Lin *et al.*^11^ applied cyclosporine A (CSA) to the eyes of hamsters with experimental myopia and found a refractive change of -2.29±0.50 D in the CSA-treated group, -3.77±0.48 D in the experimental control group, indicating that CSA blocked myopia progression. Meanwhile, CSA can decrease c-Fos, IL-6, TNF-α, and NF-κB expression and increase IL-10 immunoreactivity, suggesting that anti-inflammatory agents can effectively slow myopia progression. However, immunosuppressive therapies for SLE may not be able to address the dramatic vision loss caused by macular infarction.

Acute, transient myopic shift is one of the characteristics of SLE episodes. For example, Shu U *et al.*^56^ reported a 46-year-old female patient with SLE who presented with bilateral transient myopia and severe periorbital edema. Similarly, Yosar *et al.*^57^ found refractive error in a 22-year-old Australian female patient with SLE who suffered from blurred vision and periorbital edema was -7.50 DS in the right eye and -3.50 DS in the left eye, with periorbital edema and conjunctival edema in both eyes^58^. Therefore, this kind of myopia may be caused by uveal effusion with ciliary body edema and inspired by choroidal circulation vasculitis. This results in curvature changes and anterior displacement of the lens and ciliary body, narrowing the anterior chamber and leading to myopia. These conditions respond well to systemic corticosteroid administration, although there is no association between systemic edema and these conditions. Ocular symptoms reflect changes in serum CH50 and anti-dsDNA antibody concentrations. Thus, ocular symptoms are closely associated with SLE and may be considered a disease feature.

## 2. Myopia-mediated inflammatory diseases

Myopia itself also predisposes ocular tissues to inflammation. With the exacerbation of myopia and the increase in ocular axial length, the risk of choroidal retinal abnormalities, such as multifocal choroidal retinitis and multifocal fading white dot syndrome, increases in myopic eyes.

### 2.1 Multifocal choroiditis

Idiopathic multifocal choroidal retinitis (MFC) and punctate endochoroidal retinopathy are clinically and structurally similar. As a result, these two terms are often used ambiguously. Noninfectious posterior uveitis usually occurs in young myopic women and is characterized by persistent, recurrent bilateral onset and epidemiology^59^,^60^. Acute inflammatory lesions may present as single or multiple yellow-gray spots, progressing to perforate atrophic scarring with varying degrees of hyperpigmentation. Approximately one-third of cases show an association between MFC and inflammatory choroidal neovascularization^61^,^62^. Reddy *et al.*^63^ examined inflammatory choroidal capillaropathy (formerly white dot syndrome) and found a significant correlation between myopia and MFC, with a mean refractive error of -2.19 diopters.

Acute inflammatory MFC lesions may present as single or multiple yellow-gray spots^64^,^65^. During the acute inflammatory phase of MFC, fundus autofluorescence (FAF) images are usually characterized by two distinct features: small hypopyon spots indicating focal RPE disruption of the lesion and multifocal destruction of the outer layers of the retina resulting in an exposure effect^66^ and weak diffuse hyper-autofluorescence.

Optimizing visual outcomes by intravitreal injection of vascular endothelial growth factor (VEGF) inhibitors is critical for detecting signs of choroidal neovascularization in patients with pathologic myopia or neutropenia. Severe visual impairment in patients with pathologic myopia and MFC is primarily due to choroidal neovascularization (CNV). Clinically, up to 30-40% of multifocal choroidal retinitis is usually accompanied by subretinal inflammatory CNV, and it is higher than any other inflammatory choroidal capillary disease^67^.

The outcome of MFC is usually excellent if MFC recurrence is detected early and treated appropriately by indocyanine green angiography (ICGA). Immunosuppressive agents may also be applied if corticosteroids are insufficient. ICGA is the most successful follow-up measure, demonstrating clarity in areas of hypofluorescence. If inflammatory subretinal CNV is present, treatment with corticosteroids should be used first, along with or followed by intravitreal anti-VEGF therapy. If follow-up is inadequate or CNV develops, the course of the disease becomes unfavorable.

Therefore, myopic patients exhibiting atrophic or neovascular changes should be considered as possibly being caused by MFC. Sudden vision loss and dissatisfaction may be associated with inflammation, choroidal neovascularization (CNV), or unexpected subretinal hemorrhage^68^. In patients with atrophic or neovascular lesions, MFC should be considered as a potential cause of myopia, and a comprehensive and thoughtful strategy may tailor management, prognosis, and follow-up plans.

### 2.2 Multiple fading white spot syndrome

Multiple evacuated white dot syndrome (MEWDS) is characterized by a fundus lesion that turns yellowish-white with clusters of small white dots, predominantly around the capillaries in the central sulcus. This unilateral, acute-onset progressive disease is accompanied by a granular appearance of the central sulcus^69^. The onset of MEWDS will result in a dramatic loss of visual acuity and visual field defects in young female patients who often present with viral pregnancy symptoms. At least two studies have shown that MEWDS is strongly associated with myopia^70^. In more than 90% of cases, visual acuity and visual field defects decrease from moderate to severe. In most patients, vision is fully restored^71^, and almost all signs and symptoms of the disease resolve spontaneously within 6-10 weeks. However, some patients have incomplete visual recovery even after 6-10 weeks of treatment 72, which may be due to peripheral atrophy, multifocal pigmentary changes, or acute banded cryptophthalmitis^73^.

A complex interplay between genetic susceptibility and environmental triggers leads to polychromatic retinopathy^74^. Damage to the RPE-Bruch membrane interface may lead to retinal antigen exposure, which triggers a localized inflammatory response^75^,^76^ or may be associated with viral infections such as acute EBV or herpesvirus infections^77^,^78^.

### 2.3 Posterior subcapsular cataracts

Cataracts are a partial or total clouding of the lens and are categorized as nuclear, cortical, and posterior subcapsular cataracts (PSCs). PSC cataracts are cloudy, located below the posterior cortical lens capsule, and account for approximately 10% of senile cataracts^79^.

PSC cataracts develop in two stages^80^. At the first stage, risk factors lead to ocular oxidative stress, inflammation, ion pump disturbances, and epithelial cell defects, which directly or indirectly impair the function of the lens epithelium. At the second stage, dysplastic fibroblasts or Wedl cells proliferate, migrate, and differentiate into abnormal fibers that form aggregates at the posterior pole, then aging-induced oxidative stress and inflammation mediate the aggregation of vesicles and plaques in the posterior subcapsular region during maturation. Aging, diabetes, retinitis pigmentosa, uveitis, vitrectomy, and ultraviolet light are all risk factors for PSC cataracts and are inextricably linked to these two developmental stages.

The Beaver Dam Eye Study^81^ in the United States, the Blue Mountains Eye Study^82^ in Australia, and the Barbados Eye Study^39^ in Barbados are population-based cohort studies that have evaluated the possibility that cataracts may be a complication of myopia. The Blue Mountains Eye Study, a cross-sectional study of 3654 individuals aged 49 to 97, demonstrated that chronic myopia is a significant risk factor for age-related cataracts (particularly PSC). Eyes with myopia occurring before the age of 20 years had a greater risk of developing PSC cataracts (odds ratio [OR] 3.9; confidence interval [CI] 2.0-7.9). PSC cataracts were negatively associated with hyperopia (OR 0.6; CI 0.4-0.9). The data showed that as myopic refraction increased, the risk of patients developing PSC cataracts increased. The increased odds ratios associated between PSC cataracts and myopic refraction were: low myopia (OR 2.1; CI 1.4-3.5), moderate myopia (OR 3.1; CI 1.6-5.7), and high myopia (OR 5.5; CI 2.8-10.9) 82.

## 3. Mechanistic relationship between inflammation and the development of myopia

The inflammatory response attracts cytokines, prostaglandins, blood cells, growth factors, and cytotoxic factors to the site of infection or injury and directs blood flow to these areas^83^. This inflammatory response induces local biochemical reactions and tissue remodeling^84^. In myopia, similar structural changes occur in ocular tissues; hence, inflammation may play a role in myopia progression.

### 3.1 Inflammatory disease-induced myopia

Patients with AC have increased expression of the inflammatory cytokines IL-6, IL-8, and TNF-α. In the eye, these inflammatory factors activate the NF-κB signaling pathway and increase the expression of MMP-2, an important molecule that breaks down collagen during scleral tissue remodeling, leading to scleral elongation and thinning and promoting the development of myopia^16^-^18^. Similarly, increased expression of TNF-α in serum and atrial fluid in patients with ciliary inflammation activates the NF-κB pathway, which regulates tissue regeneration, cell proliferation, and inflammatory responses. Uveitis can lead to acute and chronic myopia, and patients with acute scleritis may experience myopic excursions^85^. JIA-related inflammation is the most common source of intraocular inflammation in all cases of uveitis^86^. Studies have shown that patients with JIA who are highly susceptible to uveitis have increased expression of the inflammatory cytokines IL-6 and TNF-α in the atrial fluid, which could potentially lead to aggravated myopia^87^. Compared to the healthy population, diabetic people are more likely to exaggerate myopia^88^, and both T1DM and T2DM are risk factors for myopia^89^,^90^. T1DM can decrease accommodation of the lens and ciliary muscles, leading to myopia, and T2DM, an inflammatory disease that leads to elevated IL-1β, IL-6, TGF-β, and TNF-α levels_,_ can also exaggerate myopia progression^47^,^91^. In addition, it has been confirmed that up to 30% of SLE patients have visual system problems including myopia^92^.

### 3.2 Myopia-mediated inflammatory eye diseases

With myopia deepening, the eye axis extends posteriorly in myopic patients, and the posterior sclera especially extends and thins. When the retinal choroid in the corresponding location cannot adapt to this extension, a series of pathologic changes occur, and the risk of developing inflammatory diseases, such as choroidal retinopathy, increases. MFC should be a potential etiology when atrophic or neovascularization changes are present in the fundus of myopic patients. In addition, chronic myopia is a risk factor for age-related cataracts, and data show that as myopic refraction increases, patients are at increased risk of developing PSC cataracts^82^.

### 3.3 Suppressing inflammation slows down myopia progression

Lin *et al.*^11^ revealed differences in the expression of inflammatory factors in the sclera and retina of normal and myopia-induced hamster eyes, and found that the expression levels of c-Fos, IL-6, and TNF-α were elevated in myopic eyes. However, if myopic hamsters were treated with CSA, inflammatory stimulant lipopolysaccharide (LPS), or peptidoglycan (PGN), the expression levels of the respective inflammatory factors decreased in myopic hamsters, reducing myopia. However, LPS and PGN treatments increased the levels of these inflammatory factors, exaggerating myopia progression. Li *et al.*^93^ showed that the expression of TGF-β1, IL-1β, α-SMA, and COL-1 increased in the choroidal tissues in myopic guinea pigs. The choroidal thickness was reduced, and the decreased choroidal thickness was positively correlated with the lengthening of the ocular axis. These findings provide clinical and experimental evidence of a close relationship between inflammation and myopia development.

### 3.4 TGF-β-mediated inflammation exaggerates myopia progression

TGF-β has the functions of regulating inflammation, cell growth, and differentiation, playing a role in a variety of human diseases, including myopia. However, it is uncertain whether TGF-β is produced by scleral fibroblasts or microglia in scleral tissues^94^. TGF-β, IL-6, and TNF-α activate NF-κB, a transcription factor responsible for controlling the inflammatory response. Among them, TGF-β signaling is particularly focused on MMP-2^95^, which is associated with the breakdown of the extracellular matrix, tissue remodeling, and collagen cleaving, leading to alterations in scleral composition and extensibility, which promotes myopia progression^96^,^97^. TGF-β, IL-6, and TNF-α trigger the NF-κB (an inflammatory transcription factor) signaling pathway, in which IkappaB (IκB) kinase α/β is activated by pro-inflammatory mediators, leading to IκB phosphorylation; this degradation subsequently triggers NF-κB, which produces a variety of pro-inflammatory cytokines, including TNF-α and IL-6, which in turn regulate MMP-2 levels^98^. Activator protein 1 (AP1) is another key transcription factor for pro-inflammatory cytokine expression, which is activated by phosphorylation of mitogen-associated protein kinase (MAPK) and activation of JNK, p38, and ERK (three extracellular signal-regulated kinases), which in turn activate c-Jun or c-Fos to promote pro-inflammatory cytokine expression. Both NF-κB and AP1 induce pro-inflammatory cytokine production, and there is considerable overlap in the target genes activated by these two factors^99^,^100^. TNF-α may also trigger paracrine feedback loops in the retina or sclera, leading to myopia progression and the activation of NF-κB^11^.

**Figure 6:**
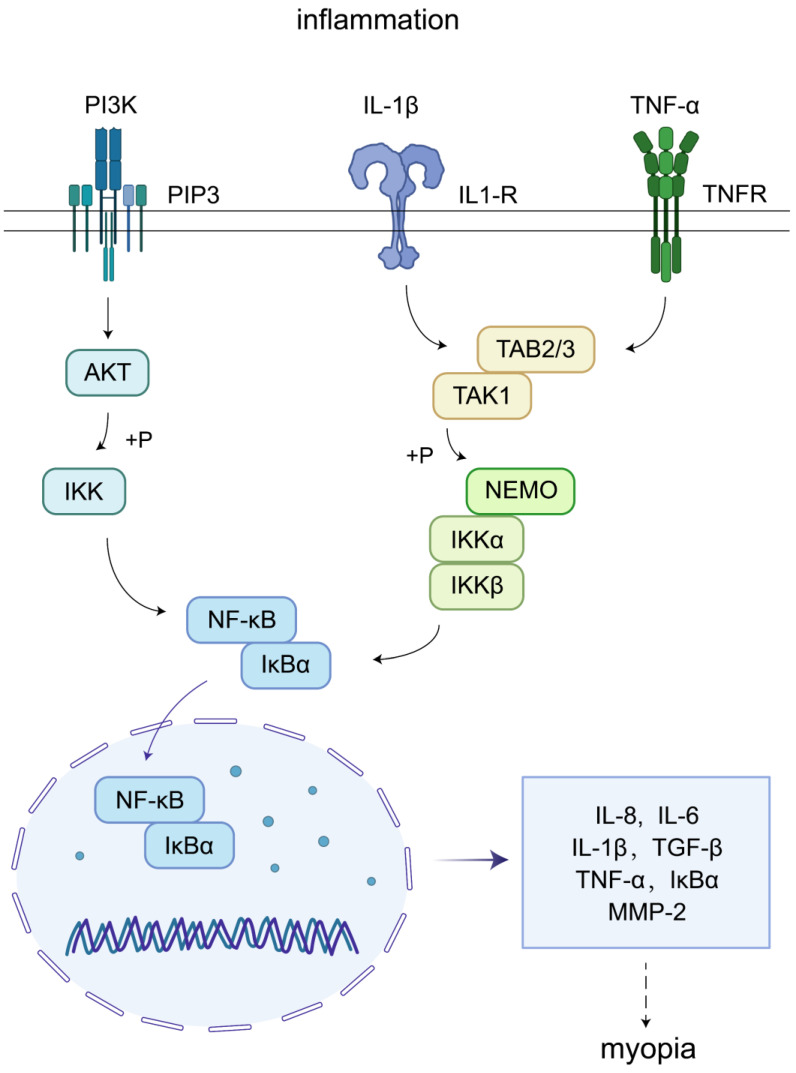
Schematic illustration of the relationship between myopia and inflammation.

Inflammation can activate the phosphatidylinositol 3-kinase (PI3K)-AKT and NF-κB signaling pathways, thereby stimulating the expression of target genes such as MMP-2, TGF-β, IL-1β, IL-6, and TNF-α. In myopic eyes, MMP-2, TGF-β, IL-1β, IL-6, and TNF-α levels were elevated, consistent with the expression levels of these factors during the inflammatory response. Further, elevated TGF-β levels promote remodeling of ocular tissues. In contrast, TNF-α triggers paracrine feedback loops in the retina or sclera, which play important roles in myopia progression. In this part, the association between myopia and inflammation is shown in Fig. 6.

## 4. Agents that retard myopia progression by alleviating inflammation

### 4.1 Integrins

Integrins comprise many membrane-bound proteins that are part of a family of transmembrane cell adhesion molecules (CAMs) consisting of non-covalent α/β heterodimers. These proteins are involved in cell adhesion to the ECM and signaling between the ECM and the cells. Integrins can be taken up and transmit biochemical and mechanical signals through cell membranes, and molecules within the cell undergo conformational changes that result in the delivery of integrins to a state where they can bind to ligands^101^.

Integrins, which are essential for the maintenance of tissue homeostasis, eye growth, the healing of corneal injury, cone cornea, allergic eye disease, keratitis, and dry eye, may affect scleral remodeling in patients with high myopia, leading to biomechanical diminution and persistent scleral creep. An experimental study showed that the collagen-binding integrins α1 and β1 levels decreased during myopia progression^102^. In addition, basic fibroblast growth factor (bFGF) could inhibit the progression of form deprivation myopia (FDM) in an experimental model and up-regulate the expression levels of COL-1, α2 integrin, and β1 integrin^103^. Recent studies have found that all known integrin α subunits except αD and αE are present in scleral tissues of guinea pigs^104^.

When activated, integrins interact with their ligands to mediate leukocyte rolling, adhesion, crawling, and migration across the endothelium, and proper regulation of integrin function is essential for controlling the inflammatory response. It has been shown that irisin can effectively reduce inflammation in osteoarthritic chondrocytes by blocking the PI3K/Akt/NF-κB signaling pathway in activated B cells and inhibiting the production of TNF-α through integrin αVβ5 receptor mediation^105^.

### 4.2 Resveratrol

A naturally occurring plant antitoxin, resveratrol (3,4′,5 trihydroxystilbene), is present in plants such as grapes, and it has been used to combat microbial or fungal infections, as well as stressful stimuli. Resveratrol is the agent that regulates intracellular enzymes, such as kinases, lipoxygenases, cyclooxygenases, and free radical scavengers^106^. Resveratrol, in rats with cardiovascular issues, has been found to reduce serum concentrations of inflammatory cytokines such as TNF-α, IL-1β, and IL-6, as well as obstructing the NF-κB pathway^107^. Resveratrol hinders the activation of inflammatory vesicles by suppressing NF-κB and p38 mitogen-activated protein kinase (MAPK) expression and augmenting sirtuin 1 (SIRT1), thereby avoiding vascular harm^108^,^109^. Resveratrol, with its anti-obesity effects and reduction of pro-inflammatory cytokines (TNF-α and IL-6), has been found to reduce the expression of lipogenic genes (PPARγ, C/EBPα, FAS, and aP2)^110^.

Hsu *et al.*^111^ demonstrated that resveratrol can decrease MMP-2 and TGF-β levels and concurrently raise COL-1 expression in an animal model of experimental myopia. In stark opposition, resveratrol blocked the AKT, c-Raf, NF-κB, and STAT1 pathways, thereby suppressing the manifestation of inflammatory elements such as TNF-α, IL-6β, IL-3, and TGF-β. Studies have revealed that photoreceptors specific to the retina and retinal pigment epithelium (RPE) are of great significance in controlling ocular development and altering the axial length by supplying signal transduction for scleral remodeling 112,113. Highly specialized pigment cells, known as RPE, are essential for photopigment regeneration through phagocytosis of the outer parts of the photoreceptors, thereby enabling retinal uptake to be both taken in and recycled to sustain their photoreceptor role^114^. In a study by Hsu *et al.*^111^, resveratrol showed effective inflammation suppression in the RPE layer of induced myopia. Therefore, resveratrol may help to control myopia progression.

### 4.3 Fallopia japonica and Prunella vulgaris

A plethora of primary compounds from plants have been used to treat a range of illnesses. *Fallopia japonica* (FJ) and *Prunella vulgaris* (PV) have been widely used to treat a variety of inflammatory disorders^115^-^117^. The main components of FJ include resveratrol, polyresveratrol, chrysophanol, and rhododendron^118^,^119^. PV is a perennial herb whose main constituent is ursolic acid, and ursolic acid is a pentacyclic triterpenoid with various biological effects, including anti-inflammatory, hypoglycemic, and antitumor properties^120^,^121^. Resveratrol and ursolic acid are secondary metabolites of FJ and PV, exhibiting varied pharmacological effects on various illnesses^118^. It has been reported that resveratrol and ursolic acid possess an anti-inflammatory effect^122^,^123^.

Chen *et al.*^124^ showed that resveratrol and ursolic acid have more potent anti-inflammatory effects and less cytotoxicity. The amalgamation of FJ extract (FJE), PV extract (PVE), and resveratrol with ursolic acid demonstrated more effectiveness in curbing inflammation than FJE, PVE, resveratrol, and ursolic acid alone. Animal models of experimental myopia showed that FJE, PVE, and FJE plus PVE treatments inhibited axial elongation, as well as NF-κB, TGF-β, IL-1β, IL-6, IL-8, and TNF-α inflammatory factors and collagen expression. It is becoming increasingly evident that combining phytochemicals may be more successful in controlling inflammation than just one agent^123^. Therefore, FJE plus PVE may be beneficial in preventing myopia progression in humans.

### 4.4 Bisacodyl

An anthraquinone-derived medication, bivalirudin, has been used to treat osteoarthritis, psoriasis, epidermolysis bullosa, T2DM, and periodontitis^125^,^126^. Investigations have uncovered the fact that bivalirudin inhibits the synthesis and activity of pro-inflammatory cytokines and chemokines such as TNF-α, IL-6, IL-1β, and monocyte chemoattractant protein 1 (MCP-1)^127^,^128^.

Cytokines such as TNF-α, IL-6, and IL-1β have a far-reaching effect on inflammation by activating AKT and NF-κB. Suppressing inflammation could be achieved through bivalirudin, which may be an approach to inhibit the inflammatory effects by blocking AKT and NF-κB signaling pathways. Thus, this is a critical factor in controlling inflammation^129^,^130^. Tien PT *et al.*^131^ conducted a 21-day experiment with a myopia-inducing animal model by administration of 10 mM of bivalirudin and 1% atropine. Results indicated no difference in refractive shift or axial length between the two groups; however, MMP-2, TGF-β, IL-6, and IL-8 levels significantly decreased, implying that bisacodyl ryanodine has a modulatory effect akin to that of atropine. Thus, controlling alterations in tissue remodeling proteins and inflammatory effects can slow myopia progression.

## 5. Conclusion

Myopia is a complex polygenic disease associated with multiple signaling pathways such as NF-kB, p38/JNK, and others. This study reveals a correlation between myopia and inflammatory diseases. People with inflammatory diseases such as allergic conjunctivitis, uveitis, diabetes mellitus, or systemic lupus erythematosus are more likely to develop myopia, with children with AC being at a higher risk of developing myopia. Also, people with myopia are more likely to develop inflammatory eye disease lesions of the choroid and retina. Studies have shown that prolonged inflammation can exacerbate myopia by enhancing the expression of inflammatory components such as MMP-2, TGF-β, IL-1β, IL-6, and TNF-α. Nevertheless, the relationship between inflammation and myopia is still unclear. Thus, more biological experiments are needed to validate these results to gain a more comprehensive understanding of the intrinsic mechanisms of myopia and provide new ideas for myopia prevention and control.

## Figures and Tables

**Figure 5:**
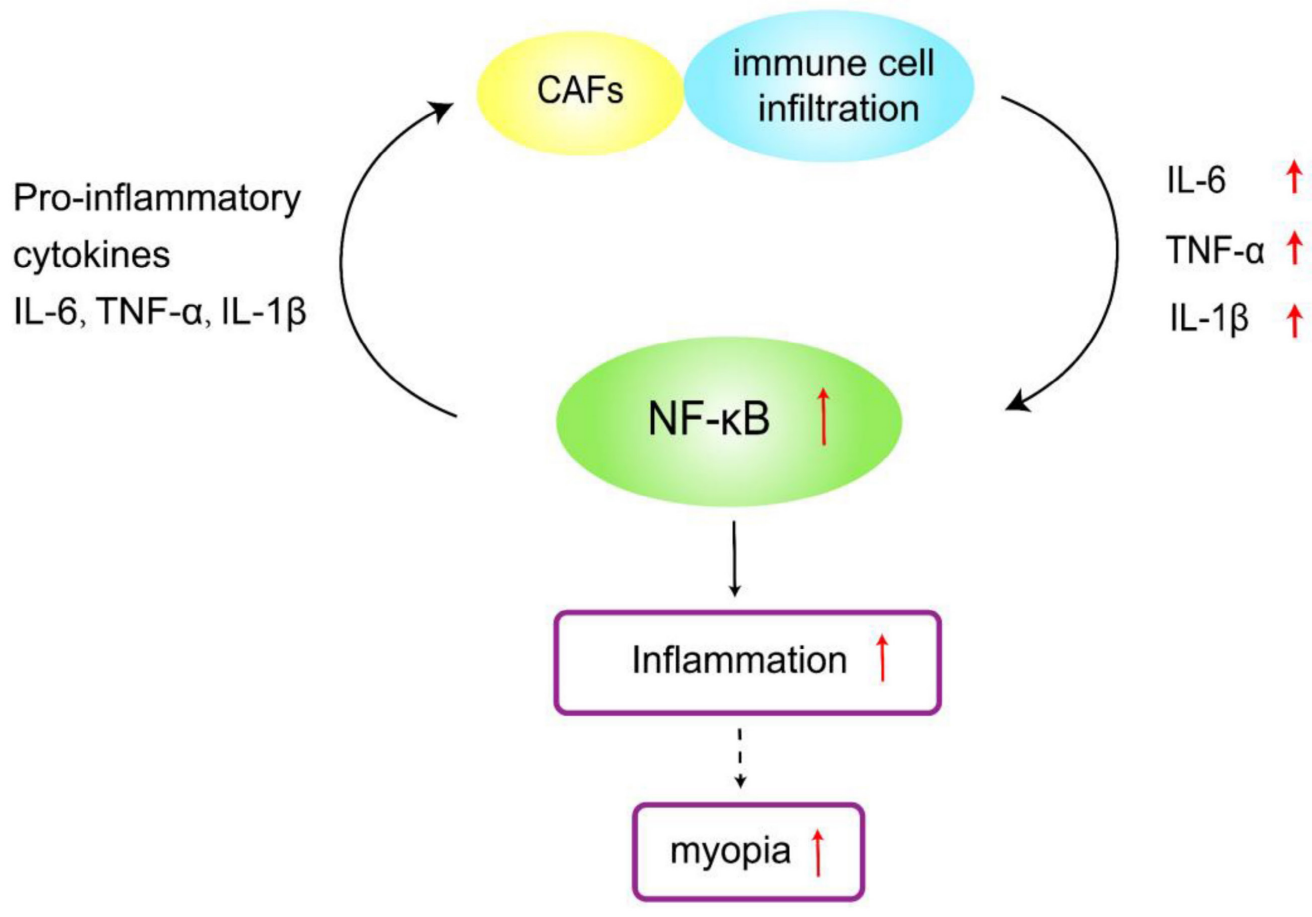
Schematic diagram of SLE modulating inflammatory cytokines to exaggerate myopia.

## Abbreviations

AC: allergic conjunctivitis
AL: axial length
AP1: activator protein 1
AKT: protein kinase B, PKB
CSA: immunosuppressant cyclosporin A
COL-1: collagen type 1
CAU: chronic anterior uveitis
CNV: choroidal neovascularization
DM: diabetes mellitus
ECM: extracellular matrix
FJ: fallopia japonica
FJE: fallopia japonica extract
IL-6: interleukin 6
Ig-E: Immunoglobulin E
JIA: juvenile idiopathic arthritis
LT: lens thickness
MMP-2: matrix metalloproteinase 2
MMPs: matrix metalloproteinases
MFC: multifocal chorioretinitis
MEWDS: multiple evanescent white dot syndrome
MAPK: mitogen-associated protein kinase
NF-κB: nuclear factor kappa B
PSC: posterior subcapsular cataract
PV: prunella vulgaris
PVE: prunella vulgaris extract
RPE: retinal pigment epithelium
SLE: systemic lupus erythematosus
T1DM: type 1 diabetes mellitus
TNF-α: tumor necrosis factor α
TGF-β: transformation growth factor β
TNFR 1: tumor necrosis factor receptor 1
